# Appropriate use of colistin in neonates, infants and children: Interim guidance

**DOI:** 10.4102/sajid.v38i1.555

**Published:** 2023-12-19

**Authors:** Vindana Chibabhai, Adrie Bekker, Marianne Black, Despina Demopoulos, Angela Dramowski, Nicolette M. du Plessis, Veshni Pillay-Fuentes Lorente, Trusha Nana, Helena Rabie, Gary Reubenson, Reenu Thomas

**Affiliations:** 1 Division of Clinical Microbiology and Infectious Diseases, Faculty of Health Sciences, University of the Witwatersrand, Johannesburg, South Africa; 2 Department of Microbiology, National Health Laboratory Service, Johannesburg, South Africa; 3 Department of Paediatrics and Child Health, Faculty of Medicine and Health Sciences, Stellenbosch University, Cape Town, South Africa; 4 Department of Microbiology, Lancet Laboratories, Johannesburg, South Africa; 5 Department of Paediatrics, Donald Gordon Medical Centre, Johannesburg, South Africa; 6 Department of Paediatrics and Child Health, Faculty of Health Sciences, University of Pretoria, Pretoria, South Africa; 7 Division of Clinical Pharmacology, Department of Medicine, Faculty of Medicine and Health Sciences, Stellenbosch University, Cape Town, South Africa; 8 Department of Paediatrics and Child Health, Faculty of Health Sciences, University of the Witwatersrand, Johannesburg, South Africa; 9 Department of Paediatrics and Child Health, School of Clinical Medicine, Faculty of Health Sciences, University of the Witwatersrand, Johannesburg, South Africa; 10 Department of Paediatrics and Child Health, Christ Hani Baragwanath Academic Hospital, Johannesburg, South Africa

**Keywords:** paediatrics, neonatology, microbiology, infectious diseases, colistin, polymyxin, carbapenem, extensively drug resistant, multidrug resistant, Gram-negative

## Introduction

This document provides interim recommendations for appropriate colistin use (polymyxin E) to treat bacterial infections in neonates, infants, and children.

Colistin, a polymyxin antibiotic, was first used clinically in the 1950s. Colistin is commercially available in two forms – colistin sulphate (oral or topical powder) and colistimethate sodium (CMS) (parenteral formulation).^1^ Colistin acts upon the Gram-negative bacterial outer cell membrane by disrupting magnesium and calcium ions. The disturbance increases cell permeability, resulting in leakage of cell contents, and ultimately cell death.^1,2^ Colistin resistance can be intrinsic or acquired. Gram-negative bacteria with intrinsic resistance to colistin include *Serratia marcescens, Proteus* spp., *Morganella* spp., *Providencia stuartii*, and *Burkholderia cepacia* complex.^3^ Acquired resistance can be chromosomal or plasmid-mediated.

In South Africa, colistin is commonly available as vials containing 1-million international unit (IU) of CMS powdered for reconstitution. Colistimethate sodium is a prodrug, which is converted in the plasma to active colistin. Each 1-m-unit of CMS vial contains approximately 34 mg colistin base activity (CBA), or 80 mg CMS. Package insert should always be consulted for quantitative vial composition.

Antimicrobial resistance (AMR) is a global public health and One-Health problem, and is considered to the next great global challenge.^4^ By 2050, an estimated 10m deaths per year will be attributable to AMR.^5^ More recently, infections with AMR pathogens caused around 1.2m deaths globally and was associated with 4.95m deaths globally in 2019.^6^

The most concerning pathogens are those listed by the World Health Organization (WHO) as priority pathogens. Among these, those categorised as critical pathogens are all Gram-negative bacteria. The latter include extended spectrum β-lactamase producing Enterobacterales, carbapenem-resistant Enterobacterales (CRE), carbapenem-resistant *Pseudomonas aeruginosa*, and carbapenem-resistant *Acinetobacter baumannii*.^7^ Research and development (R&D) of new antimicrobial agents for these pathogens is critical.

Although all age groups are at risk of AMR infections, children under the age of 5 are particularly vulnerable, with 1 in 5 deaths attributable to AMR occurring in this age group. Children living in sub-Saharan Africa are particularly affected by AMR pathogens compounded by a lack of access to effective antimicrobials.^6^

The emergence of extensively drug resistant (XDR) pathogens (an isolate that is non-susceptible to at least one agent in all but two or fewer antimicrobial categories) has resulted in renewed use of colistin.^1,8^ Several guidelines address optimal use of colistin in adults, but these do not adequately address using colistin in neonates and children.^2,9^ This document aims to provide practical guidance to clinicians for appropriate use of colistin in neonates, infants, and children. Table 1 presents a summary of the recommendations.

**TABLE 1:** A summary of recommendations.

Question	Recommendation	Strength of recommendation	Quality of evidence
What are the indications for colistin use?	Invasive infections caused by extensively drug resistant (XDR) Gram-negative organisms, predominantly, carbapenem-resistant Gram-negative infections, particularly *Acinetobacter* spp., *Pseudomonas* spp., *Klebsiella* spp., and other Enterobacterales. These are typically hospital-acquired infections.	Strong	Moderate
What is the recommended method for testing and reporting colistin minimum inhibitory concentration (MIC) results?	Broth microdilution (BMD) is the only reliable method for determining colistin MICs.	Strong	Moderate
What is the recommended colistin pharmacokinetic/pharmacodynamic (Pk/Pd) target for efficacy?	Area under the concentration time curve (AUC)/MIC is the recommended Pk/Pd parameter for colistin efficacy.	Strong	Moderate
Is an intravenous (IV) loading dose (LD) required when initiating therapy with colistin in neonates, infants and children? What LD and maintenance dose should be recommended in patients with normal renal function?	All neonates, infants and children requiring colistin should receive a colistin LD of 4 mg – 5 mg colistin base activity (CBA)/kg body weight (118 000 IU/kg – 150 150 IU/kg) Maintenance doses of colistin 2.5 mg CBA/kg/dose 12 hourly in children, infants and neonates (74 000 IU/kg)	Strong	Moderate
What maintenance dose is recommended in patients with impaired renal function and in those receiving renal replacement therapy (RRT)?	Conclusive recommendations on dosage and/or dose interval in patients with renal impairment cannot currently be established	Weak	Low
Should colistin be administered, by bolus or by infusion?	Colistin may be administered as a slow bolus injection or as a slow infusion over 30 min.	Strong	Moderate
When treating an invasive infection with colistin, is monotherapy or combination therapy recommended?	Carbapenemase producing Enterobacterales (CPE)/CRE: Combination therapy with a second active antibiotic is recommended for severe sepsis/in septic shock	Strong	Moderate
	Multidrug resistant (MDR)/ XDR *A. baumannii*: Combination therapy with a second active antibiotic is recommended for severe sepsis or in septic shock. If a second active agent is not available, colistin monotherapy is recommended.	Strong	Low
	MDR/XDR *P. aeruginosa*: Combination therapy with a second active antibiotic is recommended in those patients with severe sepsis/septic shock	Strong	Low
In which patients is empiric colistin therapy recommended?	For most patients, empiric colistin use is strongly discouraged. Empiric therapy with colistin can be considered in critically ill patients in centres where invasive infections caused by carbapenem-resistant Gram-negative pathogens are prevalent (>15% of GNB infections demonstrating carbapenem resistance)	Weak	Low
Should inhaled colistin be used to treat hospital-acquired pneumonia (HAP)/ventilator-associated pneumonia (VAP)?	Inhaled colistin, in addition to systemic colistin, using small particle nebulisers, may be considered for VAP caused by XDR Gram-negative bacteria where the pathogen is only susceptible to colistin and if there is treatment failure with systemic colistin alone.	Weak	Low
Should intraventricular/intrathecal colistin be used to treat meningitis?	Intraventricular/intrathecal colistin with IV colistin can be considered in patients with suitable indwelling devices and clinical/microbiological indications for colistin therapy	Weak	Low
How should patients receiving colistin be monitored for adverse events and how frequently?	Monitoring of renal function, sodium, potassium and magnesium, with dosage adjustments when necessary. ICU patients requiring organ support require more frequent monitoring but in patients not requiring organ support, regular monitoring at least once every 72 h. Should be clinician directed. Collect baseline prior to colistin initiation (do not delay initiation/LD while awaiting results) Daily clinical monitoring for neurotoxic side effects is recommended.	Strong	Moderate
What is the recommended duration of colistin therapy?	Meningitis- Gram-negative meningitis typically treated for 21 days VAP: typically treated for 5–7 days Urinary tract infection (UTI): typically, 3–5 days Bacteraemia: Typically treated for 7 days. (Consult microbiologist/ neonatologist/infectious diseases specialist if inadequate clinical response, and considering prolongation of colistin therapy) Intra-abdominal infection/NEC (necrotising enterocolitis): 4–8 days	Moderate	Moderate
What antimicrobial stewardship (AMS) tools are recommended when prescribing and administering colistin to patients?	An AMS ‘bundle’ to control and monitor colistin use should be implemented	Strong	Moderate

ICU, intensive care unit.

## Methods

A panel of clinical microbiologists, paediatric infectious diseases specialists, neonatologists and clinical pharmacologists was convened, representing the National Health Laboratory Service (NHLS), the South African Paediatric Association (SAPA), the United South African Neonatal Association (USANA), the Federation of Infectious Diseases Societies of Southern Africa (FIDSSA), and the South African Society of Paediatric Infectious Diseases (SASPID) to develop consensus guidance and interim recommendations for colistin use in neonates, infants, and children.

Through teleconference meetings, important concepts related to colistin use in neonates, infants and children were discussed. These topics were then assigned to panel members. Follow up meetings were held where existing evidence of colistin use in neonates, infants, and children was presented. The literature review consisted of studies published before end December 2021. Only studies published in English were included.

Thereafter, the panel generated and agreed upon a list of questions, which the guidance document aimed to answer. Panel members were divided into groups to address each clinical question. Each panel member reviewed and evaluated the literature and proposed recommendations with a brief summary of evidence.

The draft recommendations were then reviewed by the full panel. Divergences among panellists’ views were resolved through internal discussion. The finalised draft was reviewed by the USANA, the SAPA and the FIDSSA for endorsement.

Because of the scarcity of published literature in this patient population, grading of evidence, although difficult, was included.

Strength of recommendations:

- Strong: high level of confidence that there are strong benefits from this recommendation.
- Moderate: relative confidence for this recommendation.
- Weak: little confidence in the beneficial effects of this recommendation.

Quality of evidence:

- High: includes randomised controlled trials, systematic reviews, and meta-analyses.
- Moderate: non-randomised trials, cohort studies, case-control studies or diagnostic accuracy studies.
- Low: evidence based on clinical experience or expert opinion.

## Definitions

Definitions of severe sepsis and septic shock are included here, but the reader is recommended to refer to the Surviving Sepsis Guidelines for detailed definitions.^10^

Invasive infections: although this is not a comprehensive list, examples include bacteraemia, meningitis, necrotising enterocolitis (NEC)/intra-abdominal infection, bacteraemic pneumonia, bone and joint infections.

Non-severe infections: include urinary tract infection (UTI), superficial skin or soft tissue infection.

Severe sepsis:

- ≥ 2 age-based systemic inflammatory response syndrome (SIRS) criteria
- Confirmed or suspected invasive infection
- Cardiovascular dysfunction, acute respiratory distress syndrome (ARDS), or ≥ 2 non-cardiovascular organ system dysfunctions.^10^

Septic shock: the subset of severe sepsis patients with cardiovascular dysfunction, which includes hypotension, treatment with a vasoactive medication, or impaired perfusion.^10^

Critically ill: a patient with a severe airway, breathing or circulatory problem, or acute deterioration of conscious state.^11^

## Recommendations

### What are clinical and microbiological indications for colistin use?

#### Directed/targeted colistin therapy

Colistin is not active against any Gram-positive or anaerobic bacteria. It is primarily used for invasive infections caused by XDR infections, particularly *Acinetobacter* spp., *Pseudomonas* spp., *Stenotrophomonas* spp., *Klebsiella* spp., and other Enterobacterales. These are typically hospital-acquired infections.

It is also not active against some Gram-negative bacteria for example *S. marcescens, Proteus* spp., *Morganella* spp., *P. stuartii* and *B. cepacia* complex.

In general, colistin should be initiated based on confirmed or presumptive identification of an invasive XDR Gram-negative infection where no other suitable agent is available. Where possible, treatment with colistin should be in consultation with neonatologist and/or infectious diseases subspecialist. Presumptive identification refers to scenarios where the precise identification and antimicrobial susceptibility profile of an invasive isolate is underway and the patient is in a unit where carbapenem-resistant Gram-negative pathogens are prevalent.

Colistin should not be used for non-invasive or superficial infections or to treat colonisation. Clinical signs and symptoms accompanied by identification of an XDR Gram-negative pathogen from a normally sterile site (e.g. blood, cerebrospinal fluid, synovial fluid, serosal fluid) is sufficient justification for colistin treatment. Conversely, identification of such a pathogen from other, not normally sterile sites (e.g. tracheal aspirate, sputum, skin swab, stool) generally should not prompt colistin treatment unless there is compelling clinical, radiological or laboratory evidence suggesting invasive disease – such cases should be discussed with a clinical microbiologist, neonatologist and/or infectious diseases subspecialist before colistin is initiated.

#### Empiric colistin therapy

See the following recommendations regarding empiric use of colistin

### What is the recommended method for testing and reporting colistin minimum inhibitory concentration results?

#### Recommendation

*Broth microdilution is the only reliable method for determining colistin MICs according to European Committee on Antimicrobial Susceptibility Testing (EUCAST).^12,13^ The Clinical and Laboratory Standards Institute (CLSI) includes broth disk elution and agar dilution on their list of approved methods. This requires specialised equipment and expertise, and laboratories unable to perform MICs using this method, should refer isolates to laboratories with capacity to perform such testing. Disk diffusion and gradient diffusion methods should not be performed, as these are unreliable methods for colistin MIC determination. Broth microdilution is the only approved method for polymyxin B*.

*When the colistin MIC is ≥ 2 mg/L* (*for the Enterobacterales and A. baumannii*), *or ≥ 4 mg/L for P. aeruginosa, an alternative agent should be considered, as bactericidal levels are unlikely to be achieved for organisms with MICs in excess of these cut-offs. Consultation with a clinical microbiologist or infectious diseases specialist is advised to assist with appropriate interpretation of colistin MICs dependent on which antimicrobial susceptibility testing committee guideline (EUCAST or CLSI) is followed by the local laboratory*.

#### A summary of evidence

The CLSI and EUCAST are the two organisations that provide susceptibility breakpoints for colistin.

Challenges in the setting of breakpoints for colistin include: (1) difficulties in providing a reproducible MIC below 2 mg/L because of challenges in the test system; (2) in vivo levels of 2 mg/L required for bactericidal action, which is very difficult to obtain in patients with normal renal function; (3) MIC distribution data indicates that 2 mg/L is the most reliable cut-off point to separate wild type isolates from those with resistance mechanisms for the Enterobacterales and *A. baumannii*, but higher (4 μg/L) for *P. aeruginosa*.^14^

The current (2022) CLSI guidelines have set colistin breakpoints for Enterobacterales, *P. aeruginosa*, and *Acinetobacter* spp at ≤ 2 mg/L (intermediately susceptible) and ≥ 4 mg/L (resistant), with no susceptible category (Table 2).^12^ The CLSI definition of the intermediate category implies uncertainty related to susceptibility testing, clinical outcome, dosing, and administration.

**TABLE 2:** Colistin breakpoints in mg/L as per Clinical and Laboratory Standards Institute and European Committee on Antimicrobial Susceptibility Testing.

Organism	CLSI		EUCAST	
	Intermediate ≤	Resistant >	Susceptible ≤	Resistant >
Enterobacterales	2	4	(2)	(2)
*Acinetobacter baumannii* complex / species	2	4	(2)	(2)
*Pseudomonas aeruginosa*	2	4	(4)	(4)

CLSI, Clinical and Laboratory Standards Institute; EUCAST, European Committee on Antimicrobial Susceptibility Testing.

In contrast, EUCAST has maintained the susceptibility category, for *A. baumannii* and the Enterobacterales at ≤ 2 mg/L, and resistant if > 2 mg /L. *Pseudomonas aeruginosa* has slightly higher breakpoints (susceptible if ≤ 4 mg/L and resistant if > 4 mg/L) (Table 2).^13^

European Committee on Antimicrobial Susceptibility Testing introduced brackets for the colistin breakpoint. This is to warn against using colistin without additional therapeutic measures. Breakpoints in brackets represent the epidemiological cut-off value (ECOFF), which distinguishes isolates with and without acquired resistance mechanisms.^13^

### What is the recommended colistin PK/PD target for efficacy?

#### Recommendation

*Area under the concentration time curve (AUC)/minimum inhibitory concentration (MIC) (fAUC/MIC) is the recommended PK/PD parameter for colistin efficacy*.

#### A summary of evidence

An AUC_0–24_ of 50 mg.h/L is considered acceptable to achieve adequate efficacy in the presence of isolates with a MIC of ≤ 2 mg/L.^2^ Efficacy data are lacking for the paediatric population but extrapolation from adult data suggests that a steady state concentration (C_ss_) of 2 mg/L equates to an AUC_0–24_ of 50 mg.h/L.^2^ However, adult and paediatric pharmacokinetic studies have shown extensive interpatient variability in C_ss_.^15,16,17^

### Is an intravenous loading dose required when initiating therapy with colistin in neonates, infants and children? What loading dose and maintenance dose should be recommended in patients with normal renal function?

#### Recommendation

*All neonates, infants and children requiring colistin should receive a colistin loading dose of 4 mg – 5 mg CBA/kg body weight (equivalent dose in million units MU is 118 000 IU/kg – 150 150 IU/kg), which should precede maintenance doses of colistin 2.5 mg CBA/kg/dose 12 hourly in children > 2 years (equivalent dose in MU is 74 000 IU/kg). We recommend the same maintenance dosing strategy in children < 2 years until further data are available*.

#### A summary of evidence

Colistin loading doses in adult patients are recommended and well established.^2^ A colistin loading dose prevents exposure to sub-therapeutic concentrations for a prolonged period during initial treatment, as colistin concentrations rise slowly after administration.^2^

The Food and Drug Administration (FDA) and the European Medicines Agency (EMA) currently suggests no paediatric loading dose with a maintenance colistin dose of 2.5 mg to 5 mg CBA/kg per day.^18,19^ The reason for no loading dose is mainly because of a lack of robust PK and safety data in the paediatric population. Recent PK studies in children and neonates have however shown that the FDA- and EMA-recommended colistin doses are insufficient for optimal efficacy according to recommended Pharmacokinetics (PK), pharmacodynamics (PD) parameters.^15,20,21^ To our knowledge, there is only report on the pharmacokinetics of colistin in neonates.^20^ This was a prospective, open label study, performed in neonates (5–15 days of life) receiving colistin within a neonatal intensive care unit.^20^ Further elaboration on loading dose is available in the supplementary document.

In addition, paediatric colistin studies reported reversible nephrotoxicity in 3% – 10% of patients,^17^ but the impact of immature neonatal metabolic and renal function on the metabolism and elimination of colistin has not been assessed.

Based on evidence that the standard 5 mg CBA/kg per day colistin doses without loading doses produce suboptimal exposures,^22,23^ and given the emerging paediatric and neonatal PK data of colistin showing benefits of a colistin loading dose and likely subtherapeutic concentrations with the suggested maintenance dose, we recommend a colistin loading dose of 4 mg – 5 mg CBA/kg body weight, followed by 2.5 mg CBA/kg twice daily maintenance dose in neonates, children, and infants. This should be performed with close monitoring of renal function (see ‘How should adverse effects be monitored and how often’).

It is important to observe that therapeutic drug monitoring to guide colistin dosing is not available in South African at the time of publication.

### What maintenance dose is recommended in patients with impaired renal function and in those receiving renal replacement therapy?

#### Recommendation

*Data on colistin pharmacokinetics in the paediatric population with renal impairment are lacking. Conclusive recommendations on dosage and/or dose interval in patients with renal impairment cannot be established. Where alternative treatment options are available, colistin should be avoided. Where no alternative is available, individual cases should be discussed with a clinical pharmacologist, microbiologist or paediatrician to assist with dose adjustment*.

#### A summary of evidence

Overall, fewer paediatric patients experience colistin-associated acute kidney injury than adults.^22^ Colistin renal dose adjustments in adults are well established but are lacking for the paediatric population.^2,18^ Most paediatric studies evaluating the pharmacokinetics of colistin exclude patients with renal impairment.^20,23,24^ A recent population pharmacokinetic study of colistin explored covariates influencing target attainment in a population with a median age of 2.6 years (interquartile range [IQR] 0.8–6.8 years). Creatinine was a significant covariate and colistin dosing adjustments are proposed in impaired renal function. However, the proposed dosage changes have not been validated.

The pharmacokinetics of colistin in paediatric patients receiving renal replacement therapy (RRT) have not been adequately evaluated. No recommendations can be made concerning colistin dosing in patients receiving RRT. In adult patients, dose adjustments are recommended depending on the type of RRT received.^2^ In the absence of data, colistin dosing regimens during RRT should be selected through a multidisciplinary team approach, including neonatologists, nephrologists, microbiologists, clinical pharmacologists, infectious disease specialists, and chemical pathologists.

### How should colistin therapy be administered?

#### Recommendation

*Colistin may be administered as a slow bolus injection or as a slow infusion over 30 min*.

#### A summary of evidence

The contents of the vial can be reconstituted and administered as a slow bolus injection over 3 to 5 min or as an infusion over 30 min.^18,19^ The volume chosen for infusion should be determined by patient’s fluid requirements. Table 3 is a CMS conversion table.^18^ Refer to Online Appendix 1 for a practical, stepwise approach to administering CMS.

**TABLE 3:** Colistimethate sodium conversion table adopted from electronic medicines compendium.

≈ mass of CMS (mg)†	Potency	
	IU	≈ mg CBA
1	12 500	0.4
12	150 000	5
80	1 000 000	34
360	4 500 000	150
720	9 000 000	300

*Source*: From Pharmaceuticals B. Colistimethate sodium 1 million IU powder for solution for injection [homepage on the Internet]. 2018, pp. 1–10. [cited 2023 Jan 26]. Available from: https://wwwmedicinesorguk/emc/product/5648 ^18^

CBA, colistin base activity; CMS, colistimethate sodium; IU, international unit.

† , Nominal potency of the drug substance = 12 500 IU/mg.

### When treating an infection with colistin, is monotherapy or combination therapy recommended?

#### Recommendation for carbapenemase producing enterobacterales (CPE)/carbapenem resistant enterobacterales

*Combination therapy with a second active antibiotic is recommended in those patients with severe sepsis or septic shock at presentation, bloodstream infection (BSI) with a non-urinary/non-biliary source of infection or severe underlying disease. Factors for consideration when selecting the second active antibiotic* (*meropenem, tigecycline, aminoglycoside, other*) *include the site*(*s*) *of infection, the antibiotic MICs and patient renal function. High dose prolonged infusion meropenem can be used when the MIC is ≤ 8 mg/L*.

*For non-severe infections* (*e.g. uncomplicated UTI*) *colistin monotherapy can be considered in the absence of other appropriate treatment options*.

#### A summary of evidence

Several factors support combination therapy: in vitro data showing synergy of antibiotic combinations, lower efficacy of colistin monotherapy compared with β-lactam monotherapy, the potential to reduce mortality in severely ill patients, lower risk of resistance development (e.g. against colistin), and shorter treatment duration. However, several facts argue against combination therapy: the possible rise in resistance rates because of an overall increase in selection pressure following greater release of antibiotics into the environment, higher rates of adverse effects (such as nephro- and ototoxicity because of colistin or aminoglycosides), increased *Clostridioides difficile*-associated infections, fungal infections, higher costs, and possible antagonism.^25^

Although data supporting combination therapy is sparse, and largely for adults with *Klebsiella pneumoniae* Carbapenemase (KPC) BSIs, recent meta-analyses concluded that combination therapy reduces mortality and improves clinical outcomes in patients with BSIs because of CPE.^25,26^

The newer β-lactam-β-lactamase inhibitor combinations are not readily accessible in many countries, resulting in limited treatment options being available for CRE infections. Available treatments include colistin, the aminoglycosides, tigecycline and PK/PD-optimised doses of meropenem. The selection of specific antibiotics must be based on the likelihood of achieving therapeutic drug levels at the site of infection (site of infection, antibiotic MIC and PK/PD parameters) without irreversible or severe adverse effects related to the drug.

Data from observational studies on combination therapy are inconsistent.^2^ Some observational studies showed benefit of meropenem-based combination therapy for CPE (mainly KPC) when the meropenem MIC is 8 mg/L or lower.^27,28^ The second agents used in these patients varied and included colistin, tigecycline and aminoglycosides. In contrast, the AIDA and OVERCOME randomised controlled trials (RCTs) did not find a significant survival benefit of colistin-meropenem combination therapy over colistin monotherapy in patients with severe CPE infections.^29,30^

Other studies show a benefit of combination therapy only with severe sepsis or septic shock at presentation, BSI with non-urinary/non-biliary source of infection, or severe underlying disease.^31,32^ A systematic review and meta-analysis comparing monotherapy to combination therapy for MDR Gram-negative infections demonstrated reduced mortality in BSIs and in infections caused by CPEs when combination therapy comprising two active antibiotics was used.^25^ There was no reduction in mortality when combination therapy included only one active antibiotic. Clinical cure rates with mono- and combination therapy were the same. Overall, the quality of studies included in this analysis was low. The improved outcomes observed when colistin is combined with other antibiotics may be because of suboptimal pharmacokinetic properties of colistin.

Based on the available evidence, combination therapy is recommended in patients with CPE infections who have severe sepsis or septic shock at presentation, BSIs with a non-urinary/non-biliary source of infection or severe underlying disease.

#### Recommendation for extensively drug resistant *Acinetobacter baumannii*

*Combination therapy with a second active antibiotic is recommended for severe sepsis/septic shock. Factors to be considered when selecting the second active antibiotic* (*meropenem, tigecycline, aminoglycoside etc*.) *include the site and source of infection, the antibiotic MICs, and patient renal function. Consideration may be given to the use of high dose extended infusion meropenem when the meropenem MIC is* ≤ *8 mg/L and an alternate second active antibiotic is not available. Rifampicin is not recommended as a second antibiotic. If a second active agent is not available, colistin monotherapy is recommended. Where other antimicrobial agents with A. baumannii coverage are available* (*e.g. ampicillin-sulbactam, cefiderocol*), *these are recommended over colistin monotherapy*.

*For patients with non-severe infections* (*e.g. uncomplicated UTI, skin and soft-tissue*) *colistin monotherapy can be considered in the absence of other appropriate treatment options*.

#### A summary of evidence

The choice of antibiotic treatment should be based on susceptibility testing balancing the expected clinical success rate against the risk of development of ABR and the risk of severe side effects.^25^

The AIDA RCT compared colistin monotherapy to colistin-meropenem combination therapy in 406 patients, for severe infections (largely pneumonia and bacteraemia caused by *A. baumannii*), they however, did not find a difference in clinical outcomes.^29^ A planned sub-analysis of the subset of infections caused by organisms with meropenem MICs ≤ 16 mg/L was not possible because of low numbers. The OVERCOME RCT, which also compared colistin to colistin-meropenem combination therapy for carbapenem-resistant Gram negative pneumonia and BSI found no survival benefit with combination therapy.^30^ The XDR *A. baumannii* isolates prevalent currently have high carbapenem MICs (> 8 mg/L) (unpublished data) and the findings of the cited studies do not support the routine use of colistin-carbapenem combination therapy.

Similarly, despite in vitro studies demonstrating synergy when colistin is combined with rifampicin for *A. baumannii*, current clinical data do not support the use of colistin-rifampicin combination therapy.^33,34^

The XDR *A. baumannii* isolates prevalent in the South African public health sector currently have high carbapenem MICs (> 8 mg/L). The lowest concentration of meropenem and imipenem inhibiting 50% and 90% (MIC_50_ and MIC_90_ of 2019 paediatric bloodstream *A. baumannii* isolates for Gauteng, KwaZulu-Natal, Free State, and Western Cape provinces are > 8 mg/L (unpublished data courtesy of Prof Olga Perovic, Antimicrobial Resistance Laboratory, National Institute for Communicable Diseases [NICD]).

The evidence supporting combination therapy for *A. baumannii* is of low quality. However, the pharmacokinetic properties of colistin result in suboptimal drug levels at some infection sites when administered intravenously. This, together with its narrow therapeutic window, can result in limited clinical efficacy for many infections.^2^ In addition, delays in appropriate therapy (because of the MDR phenotype) may result in high bacterial burdens. Hence, combination therapy with a second active antibiotic is recommended in those patients with severe sepsis/septic shock. If a second active agent is not available, colistin monotherapy is recommended. For patients with non-severe infections (e.g. uncomplicated UTI) colistin monotherapy can be considered in the absence of other appropriate treatment options.

#### Recommendation for extensively drug resistant *Pseudomonas aeruginosa*

*Combination therapy with a second active antibiotic is recommended for severe sepsis or septic shock at presentation, BSI with a non-urinary/non-biliary source of infection or severe underlying disease*.

*For patients with non-severe infections* (*e.g. uncomplicated UTI*), *colistin monotherapy can be considered in the absence of other appropriate treatment options*.

#### A summary of evidence

There is little data available limiting a robust recommendation. In the small number of carbapenem-resistant *P. aeruginosa* cases included in the AIDA and OVERCOME RCTs, no survival benefit was demonstrated for colistin–carbapenem combination therapy over colistin monotherapy.^29,30^ Available data are mainly small retrospective studies that used a variety of active and non-active second antibiotic agents, and it is not possible to make conclusions about the value of colistin-combination therapy.

It is suggested that combination therapy is used in those patients with severe sepsis/septic shock and colistin monotherapy is used for non-severe infections.

### When is empiric treatment with colistin indicated?

#### Recommendation

*In authors’ view, empiric colistin therapy can be considered in critically ill patients in centres where invasive infections caused by carbapenem-resistant Gram-negative pathogens are prevalent. However, empiric colistin should be stopped as soon as possible e.g. alternative antibiotic active against isolate, clinical improvement, exclusion of invasive infection, non-infectious cause for illness identified*.

*For most patients, empiric colistin use is strongly discouraged. It is recommended that empiric use of colistin be approved through the hospital AMS committee on a case-by-cas e basis*.

*When empiric colistin treatment is being considered, it should only be initiated following consultation with a clinical microbiologist, neonatologist or infectious diseases specialist as there are frequently better alternatives available. Strict guidelines must be in place to guide the empiric use of colistin to prevent overuse of this last resort antibiotic and risk of colistin resistance* (*see AMS recommendations*).

It is controversial as to whether colistin should ever be prescribed empirically that is without confirmed or presumptive infection with a carbapenem-resistant Gram-negative pathogen.

The possible harms related to delayed initiation of potentially life-saving treatment must be weighed up against the benefits of preserving activity for this ‘last-line’ agent against highly-resistant bacteria. Table 4 outlines a step-by-step guide for prescribing colistin empirically.

**TABLE 4:** Step-by-step guide for empiric colistin use.

Risk assessment and management	Guidance	Additional information
Step 1: Determine unit epidemiology	- Consult Microbiologist for unit antibiogram – should be updated at a minimum annually	- Consider empiric use *only in* units with high prevalence of carbapenem resistant Enterobacterales (CRE), XDR *A. baumannii* or XDR *P. aeruginosa* OR in an outbreak setting
Step 2: Clinical suspicion	Clinical signs and symptoms of sepsis† in a patient admitted to hospital ≥ 48 h with rapid clinical deterioration	
Step 3: Laboratory Work-up	Blood cultures	
	PLUS cerebrospinal fluid (CSF): - all neonates - older children with signs and symptoms of meningitis	If it can be safely collected
	+ Inflammatory markers (CRP or PCT)	As per usual practice in the unit
	+ Specimens from suspected site of sepsis	Specimens from suspected site of sepsis which may include: catheter tip, urine, fluid/tissue, tracheal aspirate for microscopy, culture and susceptibility testing (MC&S)
Baseline renal function	Do not delay initiation of colistin while awaiting results
Step 4: Initial administration of colistin	Loading dose – 4 mg – 5 mg CBA/kg (150 000 IU/kg)	
	Followed by maintenance dose 2.5 mg CBA/kg (74 000 IU/kg)	Approximately 12 h after L/D
	Empiric colistin should be given *in addition to* a 2nd GNB active agent depending on local antibiogram data	Consult Microbiologist/antimicrobial stewardship pharmacist to provide local antibiogram
Step 5: Clinical Review 12–24 h after colistin initiation	If biomarkers low – repeat biomarkers – if still low, consider early cessation of colistin	Ensure adequate source control – imaging to assess for collections in the abdomen, brain, chest, remove central venous catheters, bone scans
	Biomarkers high – continue therapy	
Step 6: Follow up culture results and determine duration of therapy	Culture positive: switch to targeted therapy Culture negative + elevated biomarkers + rapid response to treatment – complete 5–7 days and stop if patient clinically stable	If CSF suggestive of meningitis, but cultures negative – discuss with Infectious diseases specialist/microbiologist
	Culture negative + low biomarkers + patient clinically unstable: Discuss management with infectious diseases specialist/microbiologist - Repeat blood culture/cultures from suspected site of sepsis - Check if source control was achieved - Look for alternate cause – consider early cessation of colistin at 48–72 h	Discuss with infectious diseases specialist

XDR, extensively drug resistant; CRP, C-reactive protein; PCT, procalcitonin.

† , As per standard definitions of the term.^40^

#### A summary of evidence

Infections with XDR Gram-negative organisms are associated with increased mortality. One possible explanation for the increased mortality seen is the likely delay in starting treatment with an appropriate empiric antimicrobial, as none of these organisms would be susceptible to the standard empiric treatment regimens used. Inappropriate empiric antibiotic therapy and delays in starting appropriate antimicrobial therapy are associated with increased mortality.^35,36,37^ A recent study looking at starting empiric colistin/imipenem in patients with severe sepsis showed a significant reduction in vasopressor requirement and faster improvement in inflammatory markers when treatment was appropriate.^38^ The choice of empiric antimicrobial therapy should be based on the local epidemiology of isolated pathogens as well as culture–site specific local antibiograms (e.g., blood culture specific antibiograms). Empiric use of colistin in units with a high burden of XDR Gram-negative bacteria (> 15% XDR) may be considered, but could lead to the overuse of this last resort antibiotic and increase the risk of colistin resistance. Therefore, strict guidelines must be in place to guide the empiric use of colistin for suspected invasive infection with XDR Gram-negative bacteria. Access to facility level data in the South African public sector is available on the NICD dashboard.^39^

### Should inhaled colistin be used to treat hospital-acquired pneumonia/ventilator-associated pneumonia?

#### Recommendation

*Inhaled colistin in ventilator-associated pneumonia* (*VAP*) *because of colistin-only susceptible Gram-negative bacteria in critically ill children is not routinely recommended*.

*However, inhaled colistin, in addition to systemic colistin, may be considered in VAP because of colistin-only susceptible Gram-negative bacteria for treatment failure by systemic colistin alone and with the availability of small particle nebulisers*.

*Although inhaled colistin may be a beneficial adjunct to IV colistin by leading to shorter time to bacterial eradication, significant differences in the clinical and microbiological outcomes of children with VAP have not been demonstrated*.

*When considering adding inhaled colistin to systemic colistin, prior consultation with a paediatric infectious disease specialist/microbiologist is recommended*.

#### Practical considerations

- Ensure optimal dosing of systemic colistin before adding inhaled colistin.
- The ideal nebuliser should be able to deliver colistin particles of < 3 μm diameter.
- Administer immediately after dissolving in 4 mL sterile isotonic saline solution over 15 min at a dose of 4 mg CBA/kg 12 hourly (120 000 IU/kg 12 hourly).
- Monitor for respiratory side effects, for example, bronchospasm.

#### A summary of evidence

The hydrophilic structure of colistin limits its penetration into lung parenchyma.^41^ Nebulised colistin potentially achieves higher concentrations in the airways with less systemic toxicity, compared with intravenous (IV) colistin.^42,43^ To reach the lung parenchyma, the particle size, expressed as mass median aerodynamic diameter (MMAD), should be about 3 μm.^44^ Most nebulisers are designed to effectively deliver drug to the airways, not the lung parenchyma, and create aerosols of 5 μm MMAD.^44^ Nebulisers able to provide sufficiently small particles (< 3 μm) to reach the lung parenchyma include jet, ultrasonic, and vibrating-mesh nebulisers.^45^ In addition, the absorption rate will depend on many factors including the location of deposition (central versus peripheral) as well as the volume and mechanical properties of airway secretions.^43^

Although inhaled colistin is widely used for cystic fibrosis (CF), the pharmacodynamics might be different in a patient without chronic lung disease.^46,47^ The bacteria in patients with CF are mainly found in the mucus rather than on the epithelial surface.^43^

The evidence for inhaled colistin in adults with VAP because of multidrug resistant (MDR) Gram-negative organisms is of low quality.^48^ Expert opinion from professional societies, such as the Infectious Disease Society of America (IDSA) and the European Society for Clinical Microbiology (ESCMID) are conflicting.^49^ The evidence for inhaled colistin in the paediatric population is limited to small retrospective studies, with no respiratory complications reported: most infants received inhaled colistin in addition to active systemic antibiotics.^50,51^ Monotherapy with inhaled colistin (without systemically administered antibiotics) was successful in 17 neonates.^52,53^ A matched case control study found that infants treated with nebulised colistin plus systemic colistin, had better outcomes than infants treated with systemic colistin alone.^54^ The addition of inhaled colistin to IV colistin led to a shorter time to bacterial eradication in critically ill children with VAP because of colistin-only susceptible GNB. However, it did not lead to a significant difference in the clinical and microbiological outcomes of VAP.^55^

A PK/PD study suggested that a dose of 4 mg CBA/kg (120 000 IU/kg) attained high colistin levels in tracheal aspirate from neonates for 12 h.^56^

Adverse effects associated with inhaled colistin are bronchospasm and nephrotoxicity. High concentrations may cause damage to the airways. Nebulised steroids and β-2-agonists can be used to prevent and treat bronchospasm.^44^

### Should intraventricular/intrathecal colistin be used to treat meningitis?

#### Recommendation

*The use of intraventricular/intrathecal colistin in combination with IV colistin can be considered in patients with suitable indwelling devices and clinical/microbiological indications for colistin therapy* (*See above recommendations for indications*). *In addition, for patients without a suitable indwelling device but persistent CNS infection despite maximum recommended IV colistin dosing, neurosurgery and microbiology/infectious diseases consultation is recommended prior to initiating intraventricular/intrathecal colistin. Proposed dosing of intrathecal/intraventricular colistin is detailed in Table 5*.

**TABLE 5:** Suggested/proposed colistin intrathecal/intraventricular dose.

Age group	Suggested dose†
Neonates	0.26 mg CBA/day or 7500 IU/day (15% of infant dose)^61^
Infants	1.7 mg CBA/day or 50 000 IU/day (40% of adult dose)
Children > 1 year	2.6 mg – 4.25 mg CBA/day or 75 000 IU IU/day – 125 000 IU/day

CBA, colistin base activity; IU, international units.

† , Dose based on estimated CSF volume – **note that this is NOT dose per kg**.

#### Summary of evidence

Colistin does not cross the blood-CSF barrier well.^57^ Intrathecal (through the lumbar thecal sac) or intraventricular (lateral ventricle) dosing of colistin bypasses this barrier and can achieves much higher CSF colistin levels than with IV dosing.

Intrathecal and intraventricular colistin administration has not been well studied.^58^ Available low quality evidence suggests possible limited benefit and no concerns of long-term/irreversible harm.^57,58.59,60,61,62^ We therefore, recommend consideration in patients with suitable indwelling devices, such as lumbar or external ventricular catheters/drains. For patients without a suitable indwelling device but persistent CNS infection despite maximum recommended IV colistin dosing, neurosurgery and microbiology/infectious diseases consultation is strongly recommended prior to initiating intraventricular/intrathecal colistin.

Ventricular volume and CSF drainage rates must be considered when selecting a dosing regimen.^63^ The CSF volume in neonates is small (approximately 5 mL) compared with that in older infants (50 mL) and adults (125 mL – 150 mL).^56^ Doses of 4 mg CBA/day (120 000 IU/day) in adult patients yields CSF through concentrations above 2 mg/L.^23,64^ The IDSA suggests that for infants, adult intraventricular antimicrobial doses should be reduced by 60% or more. A retrospective review of colistin use for MDR Gram-negative infections in a Pakistan neonatal unit, briefly described the management of 15 cases of meningitis. Seven neonates received intraventricular colistin, five of whom also received IV colistin. The five neonates who received both intraventricular and IV colistin, and one of two who received only intraventricular therapy, survived. None of the eight neonates who received only IV colistin survived. The basis for the dose selection in this unit was not provided.^61^

Individualised dosing based on predicted ventricular volume and CSF drainage is recommended. Multidisciplinary (neurosurgery, microbiology, infectious diseases, paediatrics and clinical pharmacology) consultation is recommended for dose selection.

If administering intrathecal or intraventricular colistin, a single daily dose is recommended.

### How should patients receiving colistin be monitored for adverse events and how frequently?

#### Recommendation: Nephrotoxicity and electrolytes

- *Close monitoring of renal function, sodium, potassium and magnesium, with dosage adjustments when necessary* (*See Online Appendix 1*).
- Maintain adequate hydration.
- Limit co-administration of other nephrotoxic drugs.

Definitions of acute kidney injury are detailed in Online Appendix 1. The ICU patients requiring organ support require more frequent monitoring but if not requiring organ support, monitoring every 72 h is sufficient. Ideally, monitoring should be clinician directed. Collect a specimen for baseline renal function prior to colistin initiation, but do not delay initiating colistin loading dose while awaiting results.

#### A summary of evidence

Adverse effects because of colistin have been reported since the early 1960s, mostly from adult studies, with rates as high as 50%.^65^ Reported adverse effects included nephrotoxicity and less commonly, neurotoxicity.

Data on colistin safety in the neonatal/paediatric population is limited and mostly restricted to retrospective reviews, with recent reported rates of nephrotoxicity ranging from 0% to 24%. These rates have reduced compared with older reports, attributed to improved fluid and electrolyte management in intensive care units, as well as better monitoring of renal function and reduced concomitant use of other nephrotoxic agents. The suggested mechanism of nephrotoxicity is increased membrane permeability, causing tubulopathy, influx of electrolytes and water, leading to cell oedema and lysis.^65^ Definitions used for nephrotoxicity are not standardised and include an increase in serum creatinine > 50% above baseline, a decrease in urine output below 50% of baseline or < 1 mL/kg/h, or an increase in serum creatinine of > 0.5 mg/dL (44 μmol/L).^66,67,68^ A case series reported a 19% renal toxicity rate in neonates receiving colistin.^69^ Another retrospective study in 104 children reported nephrotoxicity in 10.5% of patients receiving colistin.^66^ These children, however, were also receiving other concomitant nephrotoxic drugs and none on colistin alone developed nephrotoxicity. Another study reported nephrotoxicity in 2 of the 18 neonates treated with colistin.^67^ In other retrospective studies in neonates, including preterm neonates, colistin was well tolerated, with no reported cases of renal impairment.^70^ Electrolyte imbalances, particularly hypomagnesemia, hyponatremia and hypokalemia, are reported in those on colistin. In a retrospective study 12 neonates on IV colistin, 2 had significant hyponatremia and hypokalemia. In this study magnesium replacement was required at least once for all patients.^71^ Another case-control study that compared 47 neonates who were given colistin with 59 neonates treated with other antimicrobial agents concluded that colistin use was significantly associated with hypokalemia and hypomagnesemia.^72^

Measures used to prevent or limit nephrotoxicity include strict monitoring of renal function with dosage adjustments when necessary, proper hydration and limiting co-administration of other nephrotoxic drugs.^9^

#### Recommendations: Neurotoxicity

- Daily clinical monitoring for neurotoxicity is recommended.
- Inspection for cumulative neurotoxicity by concomitant medication which also cause neurotoxic side effects by reviewing medication chart daily.

#### A summary of evidence

Neurotoxicity is the second most common adverse effect reported with polymyxins.^73^ Manifestations of neurotoxicity include dizziness, generalised weakness, muscle weakness, facial and peripheral paraesthesia, partial deafness, visual disturbances, vertigo, confusion, hallucinations, seizures, ataxia, neuromuscular blockade and apnoea.^65^ Neurotoxicity usually develops in the first 4 days of treatment.^74^ In a neonatal study, neurotoxicity was not an obvious problem. However, the authors observe that sedation and mechanical ventilation may have affected these findings.^20^ In a systematic review of polymyxin toxicity, neurotoxicity was reported more frequently in older literature than more recently, but still old, published literature (until 2005).^65^ Neurotoxicity was reported more frequently after a loading dose was compared in a single study, but this finding was not statistically significant.^65^ Concomitant administration of colistin with curariform muscle relaxants and other neurotoxic agents must be avoided because these combinations may trigger progression to neuromuscular blockade.^65^ Where possible, concomitant use should be avoided. Where unavoidable, close daily monitoring by physical examination is necessary and daily review of the prescriptions necessary to determine the need for concomitant use. Of note, however, detecting of neurotoxic symptoms in neonates is challenging.

### What is the recommended duration of colistin therapy?

#### Recommendation

*Treatment duration is dependent on site of infection, its severity, source control attainment, and PK/PD. Duration of treatment may differ according to indication. To prevent development of resistance to colistin, duration of therapy should be as short as possible, and guided by clinical and biomarker responses. Duration of therapy should be discussed with infectious diseases specialist/microbiologist:*

- *Meningitis: Gram-negative meningitis typically treated for 21 days*.
- *VAP: typically treated for 5–7 days*.
- *UTI: typically, 3–5 days*.
- *Bacteraemia: typically treated for 7 days. Consult microbiologist/neonatologist/infectious diseases specialist if inadequate clinical response, and considering prolongation of colistin therapy*.
- *Intra-abdominal infection/NEC: 4–8 days*.

#### A summary of evidence

The optimal duration of antibiotic therapy depends on many factors. The integration of signs of resolution, biomarkers, clinical judgement, and microbiologic eradication might help to define this optimal duration in patients with life-threatening infections caused by XDR Gram-negative bacteria. It is important to observe that prolonged therapy is not required for infections caused by MDR and XDR pathogens compared with susceptible isolates of the same species.^75^

### Which antimicrobial stewardship tools are recommended while prescribing and administering colistin to patients?

#### Recommendations

- *Colistin* (*an antibiotic of last resort*) *should be reserved to treat suspected or confirmed XDR Gram-negative and/or carbapenem-resistant infections and is listed as a ‘Reserve’ antibiotic in the WHO AWaRe classification^76^*
- *Prescription and administration of colistin for inpatients should be actively managed and tightly regulated through institutional regulation of colistin use by hospital AMS programmes and/or pharmaceutical and therapeutics committees* (*PTC*) *using one/more AMS tools.^77^*
- *Antimicrobial stewardship tools for colistin may include requirement for prescription authorisation, Section 21 reporting, implementation of post-prescription dosing and duration review by an AMS team or specialist (microbiologist, infectious diseases specialist, infectious diseases pharmacist)*.^9,77^ *An AMS ‘bundle’ to control and monitor colistin use should be implemented.^78,79^*
- *At national level, surveillance of colistin consumption and resistance rates should be conducted by the Department of Health and monitored/reported by other stakeholders for example South African Antibiotic Stewardship Programme* (*SAASP*), *the NICD and/or the Ministerial Advisory Committee on Antimicrobial Resistance* (*MAC-AMR*).*^80,81,82^*
- *At hospital level, surveillance systems should be established to monitor ABR rates among key pathogens causing healthcare-associated infection* (*Enterobacterales, Acinetobacter spp and Pseudomonas spp. etc*.).

### The general principles of antimicrobial stewardship, which apply to colistin use are outlined in Table 6

#### A summary of evidence

The previous South African colistin use guideline (2016) gave recommendations for colistin prescribing and administration in adults and children but did not include AMS for colistin use in humans. Although there is plentiful guidance on AMS implementation in hospital inpatients, few guidelines address colistin stewardship specifically.^9,77,80,81,82,83^ However, many generic AMS principles remain highly relevant to AMS for colistin as listed in Table 6. Table 7 outlines recommendations for monitoring colistin use according to a ‘colistin bundle’.

**TABLE 6:** General antimicrobial stewardship principles for colistin use.

Feature		Checked
1.	Obtain high quality appropriate microbiological samples before antibiotic administration and carefully interpret the results. In the absence of clinical signs of infection, colonisation does not require antimicrobial treatment.	-
2.	Avoid the use of antibiotics to ‘treat’ fever. Investigate the root cause of fever and treat only significant bacterial infections.	-
3.	When indicated, start empiric antibiotic treatment after taking cultures, tailoring therapy to the site of infection, risk factors for multidrug-resistant infection and the local microbiology and susceptibility patterns.	-
4.	Prescribe drugs at their optimal dose, mode of administration and for the appropriate length of time, adapted to each clinical situation and patient characteristics.	-
5.	Use antibiotic combinations only in cases where the current evidence suggests some benefit.	-
6.	When possible, avoid antibiotics with a higher likelihood of promoting drug resistance or healthcare-associated infections, or use them only as a last resort. Refer to the WHO AWaRe classification for a comprehensive list of antibiotics classified as ‘watch’.^84^	-
7.	Ensure early source control by draining infected foci quickly and removing all potentially or proven infected devices.	-
8.	Always try to de-escalate or streamline antibiotic treatment according to the clinical situation and the microbiological results.	-
9.	Stop antibiotics as soon as a significant bacterial infection is unlikely.	-
10.	Do not work alone. Set up local teams with an infectious diseases specialist, microbiologist, clinical pharmacologists, hospital pharmacist, infection control practitioner or hospital epidemiologist, and comply with hospital antibiotic policies and guidelines.	-
11.	Obtain regular updates (minimum annually) of the unit antibiogram/acquisition of local epidemiologic data to guide treatment decisions.	-

**TABLE 7:** Examples of colistin bundle elements.

Bundle element	Examples
Submit a specimen for MC&S from the suspected site of infection prior to colistin initiation (ideally from a sterile site*)	*Blood culture, *cerebrospinal fluid, urine, fluid, tissue, pus, respiratory specimens
Approval to use colistin	Medical microbiology or infectious diseases authorisation prior to dispensing
Document the clinical indication and note whether therapy was empiric or targeted	Bacteraemia, meningitis, ventilator-associated pneumonia, urinary tract infection, endocarditis, osteomyelitis
For patients with a positive culture, document the microbiological indication, including organism and antibiotic susceptibility	CRE, MDR/XDR *A. baumannii*, MDR/XDR *P. aeruginosa*
Document investigations performed to look for the source of infection	Possible sources: IV lines, endocarditis, pus collections (intra-abdominal, intracranial), urinary tract, pneumonia, prosthetic material etc.
Obtain consent to use the unlicensed drug	Complete the appropriate documentation
Consider whether renal dose adjustment is needed	Use the appropriate age and gestation related norms for creatinine and appropriate formula to calculate e-GFR. Document if renal dose adjustment is required.
Provide a loading dose and document the loading dose in mg/kg	-
Document maintenance dose in mg/kg	Hospital AMS committee should develop and circulate dosing charts to guide colistin prescribers
Document if combination therapy was used and which antimicrobial agent was given	-
De-escalation should be performed promptly based on culture results and pathogen susceptibility testing, infection source and clinical response	Document duration of therapy based on clinical indication
Ensure feedback documentation for SAPHRA is completed	Carefully document adverse events and deaths occurring during colistin therapy

CRE, carbapenem-resistant Enterobacterales; XDR, extensively drug resistant; MC&S, microscopy, culture and susceptibility testing; SAPHRA, South African Health Products Regulatory Authority.

## South African Health Products Regulatory Authority form completion and application for colistin

### Summary

Colistin is not registered by the South African Health Products Regulatory Authority (SAHPRA). For its access a Section 21 application form must be submitted to SAHPRA. The form allows access to unregistered medicines under Section 21 of the *Medicines and Related Substances Act, 1965 (Act 101 of 1965)* which states:

[*T*]he Authority may in writing authorize any person to sell during a specified period to any specified person or institution a specified quantity of any particular medicine, medical device or IVD which is not registered.^85^

The form comprises six subsections: particulars of the applicant, particulars of the person, institution or company importing the unregistered medicine, particulars of the patient, particulars of the unregistered medicine for which a Section 21 application is being made, informed consent form and a progress report. This should be duly completed by the medical personnel responsible for the patient’s management and handed to the relevant pharmacist. This application is then submitted to SAHPRA by the pharmacy departments. (More information concerning the Section 21 application process can be found at https://www.sahpra.org.za/document/2-52-section-21-access-to-unregistered-medicines/).
